# A Case of Triplets

**Published:** 1889-08

**Authors:** J. D. Westervelt


### A CASE OF TRIPLETS.

BY J. D. WESTERVELT, M. D.

June 20, 1889, delivered Alice Elmore, colored, primipara, age 23, of triplets, at the seventh month. Presentation of the first was normal. The second a breech, following in twenty minutes. In twenty minutes more came the secundines with the third fœtus intact in them. I clipped the sac and extracted it. It was mummified and much flattened, and not more than half the size of either of the others—all females. Development of the third had evidently been arrested a month or more, but owing to the preservative properties of the liquor amnii, there was no observable fetor. Each fœtus was in its own separate sac, but the sacs had a common central union. Three coins placed in a tangent circle give a fair diagram of these placentæ.

